# Recognition of Signed Expressions in an Experimental System Supporting Deaf Clients in the City Office

**DOI:** 10.3390/s20082190

**Published:** 2020-04-13

**Authors:** Tomasz Kapuscinski, Marian Wysocki

**Affiliations:** Department of Computer and Control Engineering, Faculty of Electrical and Computer Engineering, Rzeszow University of Technology, W. Pola 2, 35-959 Rzeszow, Poland; mwysocki@kia.prz.edu.pl

**Keywords:** human–computer interface, computer vision, sign language recognition

## Abstract

The paper addresses the recognition of dynamic Polish Sign Language expressions in an experimental system supporting deaf people in an office when applying for an ID card. A method of processing a continuous stream of RGB-D data and a feature vector are proposed. The classification is carried out using the k-nearest neighbors algorithm with dynamic time warping, hidden Markov models, and bidirectional long short-term memory. The leave-one-subject-out protocol is used for the dataset containing 121 Polish Sign Language sentences performed five times by four deaf people. A data augmentation method is also proposed and tested. Preliminary observations and conclusions from the use of the system in a laboratory, as well as in real conditions with an experimental installation in the Office of Civil Affairs are given.

## 1. Introduction

According to the World Federation of the Deaf, there are about 70 million deaf people in the world. They cannot easily articulate words and have great difficulty in understanding written content and in expressing thoughts in writing. Sign language is their primary means of communication.

Sign language skills in the hearing community are negligible. Moreover, most technical communication systems use written or spoken languages. Therefore, deaf people face barriers in social contacts, and they find it difficult to function alone.

That is why works are undertaken to build technical devices supporting the communication of deaf people with their environment. One of the tasks that such systems must perform is automatic sign language recognition.

This work presents the recognition of Polish Sign Language (PSL) expressions used in the experimental vision-based system supporting deaf people in the office when applying for an ID card.

The main contributions of the paper are:

1. A method of processing the sequences of depth images and skeletons, acquired using the RGB-D sensor, to determine feature vectors independent of small changes in the user position and rotation,
2. Hand segmentation in the depth image by a modified version of the seeded region growing algorithm,
3. A feature vector proposal inspired by linguistic research on the so-called three main phonological features of a sign (shape, place of articulation, and movement),
4. The experimental selection of parameters for classifiers commonly used in time series recognition and evaluation of their effectiveness while recognizing the considered sign expressions,
5. The assessment of the impact of using augmented data in the training phase obtained by averaging the original sequences aligned by dynamic time warping,
6. The recognition of signed expressions taking into account division into thematic subgroups resulting from established conversation schemes,
7. The preliminary observations and conclusions from the use of the system in real conditions.

The structure of this paper is as follows. Section 2 provides the research background and relevant literature references. Section 3 defines the problem. Section 4 describes the data acquisition and processing, as well as the proposed feature vector. The classification methods are presented in Section 5. The experiments and the resulting conclusions are described in Section 6. A summary along with a proposal for further work are provided in Section 7.

## 2. Recent Works

Comprehensive overviews of the literature on sign language recognition can be found in [1,2,3,4,5,6]. The described solutions can be divided into methods using special data gloves or computer vision.

The first glove equipped with sensors transforming selected handshapes into electrical signals was patented in 1983 [7]. Since then, many solutions have been developed, where users have to wear special gloves, clothing, or other wearable sensors [8,9,10,11,12]. The advantage of using data gloves is precision. However, they restrain the user and limit his/her freedom.

As modern solutions should strive to ensure that human-computer communication occurs naturally, the use of color cameras has become the dominant trend. In the literature, there are publications on American sign language recognition (ASL), e.g., [13,14,15], Japanese (JSL), e.g., [16,17,18], German (GSL), e.g., [19], Chinese (CSL), e.g., [20,21,22], Taiwanese (TSL), e.g., [23,24], Dutch (DSL), e.g., [25,26], Australian (Auslan), e.g., [27], Polish (PSL), e.g., [28], and many others. Most often, these solutions were based on the detection of human skin color to extract the user’s hands and face  [29]. Classification was most often carried out using: hidden Markov models (HMM), e.g., [30,31,32], artificial neural networks (ANN), e.g., [33,34,35], dynamic time warping (DTW), e.g., [27,36], and other methods. Vision methods allow natural interaction and inclusion of non-manual features [37,38], but they are dependent on lighting conditions, background colors, and the user’s clothing. Therefore, such solutions work only in controlled laboratory conditions.

The solution to these problems became possible with the appearance of RGB-D cameras on the market. Depth information allows separating the background by segmenting the person as a foreground object. It is also possible to incorporate 3D features into vectors describing recognized gestures. Moreover, gestures can be accurately described using multi-dimensional data structures, the so-called point clouds [39], expressed in real-world coordinates. It is worth mentioning that some RGB-D cameras, based on the time-of-flight principle, operate even in a dark room [40]. Solutions using stereovision and multi-camera systems have been known in the literature for a long time, e.g.,  [41,42,43]. Currently, most methods use RGB-D cameras, e.g., [44,45,46,47].

Deep learning methods are also used to recognize sign languages, e.g., [48,49,50]. However, the results obtained are not as groundbreaking as in the case of static image recognition. The reason is the lack of publicly available large training datasets described using commonly accepted annotation standards [1].

Most of the works available in the literature concern the recognition of single words or simple expressions. Only a few papers describe continuous sign language recognition, e.g., [48,50]. They require solving the problem of the temporal segmentation of the incoming data stream and the non-trivial task of distinguishing meaningful gestures from unintentional hand movements. An additional difficulty is the phenomenon of co-articulation involving the deformation of the ends and beginnings of adjacent sign characters and the epenthesis effect involving adding some transitions between signs. There are also significant differences in the speed of gesture performance.

In the literature, there are descriptions of several systems that use sign language to support deaf people. Examples of such solutions are: an Internet communicator [51], an application for sending SMS [52], games for the deaf [53,54], and educational tools [55,56]. In most cases, however, emerging systems are not based on sign language recognition and, therefore, do not meet the natural interaction paradigm.

## 3. Problem Statement

The SyKoMisystem developed at the Department of Computer and Control Engineering of the Rzeszów University of Technology is a human-computer interface supporting deaf communication with an office clerk in a public institution. The signed expressions of a deaf person are observed by a camera and translated by a computer into written or spoken language for the office clerk. Selected from the database responses of the office clerk are presented to a deaf person in the form of a short movie in sign language.

The system has a modular structure and consists of sign-to-text and text-to-sign modules, as well as dictionaries of expressions adequate for the selected application. The flexible configuration of these modules enables the creation of derivative products that do not always require bi-directional translation.

The current version allows a trained deaf person to submit an application for an ID card or collect an ID card. It is possible to adapt the system to other topics in any public institution. Information boards or interactive “kiosks” can also be built using selected modules. Such devices can support deaf people at stations, in trains, buses, planes, or even supermarkets. It is also possible to create educational tools for learning sign language in the form of games. The experimental version of SyKoMi was installed in the Civil Affairs Department of Rzeszow City Hall.

## 4. Data Acquisition and Processing

**Figure 1:**
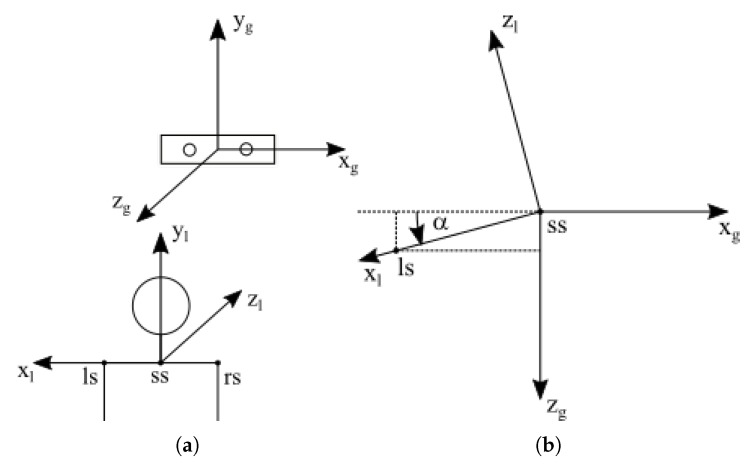
Global *g* and local *l* reference system: (**a**) illustrative view, (**b**) “top view” after translating the center of the *l* system into the center of the *g* system.

An RGB-D sensor was used, which in addition to the color and depth images, also allowed the acquisition of skeletal data. Skeletal data consisted of a hierarchical set of interconnected segments, which were bone analogs. The developed method required that the skeleton contain at least the joints corresponding to the right hand (rh), left hand (lh), left shoulder (ls), and neck (ss) (Figure 1a).

The Kinect sensor or any other RGB-D camera with dedicated software can be used to acquire such data.

The global scene reference system *g* was associated with the sensor, as shown in Figure 1. It was assumed that the deaf person sits in a chair opposite the sensor. Their hands are on their knees. Raising any hand above the plane $y=y_{hi}$ denotes the start of an expression, and lowering both hands below $y=y_{lo}$, where $y_{hi}-y_{lo}>P_{w}$ and $P_{w}$ is the palm width. The values $y_{lo}$, $y_{hi}$, and $P_{w}$ were determined experimentally. The introduction of two limits: $h_{lo}$ and $y_{hi}$, and the dead-band between them, greater than the palm width, prevented short-term state changes when the *y* coordinate of the palm oscillated around the thresholds.

The user was expected to sit in a fixed position relative to the sensor, but in fact, it was difficult to ensure this. Therefore, to become independent of minor changes in the user’s position and orientation, the coordinates of his/her hands were expressed in the local reference system *l* associated with the user (Figure 1). Using the homogeneous coordinates, we get:

(1) $$\left[\begin{array}{c} x_{l,rh}\\ y_{l,rh}\\ z_{l,rh}\\ 1 \end{array}\right]=\left[\begin{array}{cccc} -cos(\alpha ) & 0 & sin(\alpha ) & 0\\ 0 & 1 & 0 & 0\\ -sin(\alpha ) & 0 & -cos(\alpha ) & 0\\ 0 & 0 & 0 & 1 \end{array}\right]\left[\begin{array}{cccc} 1 & 0 & 0 & a\\ 0 & 1 & 0 & b\\ 0 & 0 & 1 & c\\ 0 & 0 & 0 & 1 \end{array}\right]\left[\begin{array}{c} x_{g,rh}\\ y_{g,rh}\\ z_{g,rh}\\ 1 \end{array}\right]$$

where ${\left[\begin{array}{cccc} a & b & c & 1 \end{array}\right]}^{T}$ and $\alpha =asin\left(\frac{z_{g,ls}-z_{g,ss}}{\sqrt{{\left(x_{g,ls}-x_{g,ss}\right)}^{2}+{\left(z_{g,ls}-z_{g,ss}\right)}^{2}}}\right)$ denote the translation vector and rotation angle between the *g* or *l* system and the subscripts indicate the reference system and joint name (see Figure 1). The coordinates of the left hand ${\left[\begin{array}{cccc} x_{l,lh} & y_{l,lh} & z_{l,lh} & 1 \end{array}\right]}^{T}$ in the local reference system were determined similarly.

The depth image was used to segment both hands because: (i) the use of an RGB image would involve several restrictions on the background color and clothing of the user; (ii) the depth map obtained using time-of-flight technology was more resistant to changes in lighting conditions; (iii) with sudden changes in lighting, some compensation algorithms are activated for the color stream, which reduces the frame rate and increases the blur effect; and (iv) 3D spatial information can be used to segment hands.

The shape of the hand could be determined by thresholding the depth image. However, such an approach required that it was the object closest to the camera. For some sign gestures, this is difficult to fulfill. Besides, when the user is sitting, his/her knees and thighs are closer. Therefore, an alternative solution, based on depth image segmentation by a modified version of the seeded region growing algorithm, was proposed. The initial position of the right hand $P_{d,rh}={\left[i_{d,rh},j_{d,rh}\right]}^{T}$ in the depth image reference system *d* (the seed of the algorithm) was determined based on its real-world coordinates in the *g* system and a pinhole camera model:

(2) $$\left[\begin{array}{c} wi_{d,rh}\\ wj_{d,rh}\\ w \end{array}\right]=\left[\begin{array}{ccc} \frac{1}{n_{x}} & 0 & {\tilde{d}}_{x}\\ 0 & -\frac{1}{n_{y}} & {\tilde{d}}_{y}\\ 0 & 0 & 1 \end{array}\right]\left[\begin{array}{cccc} f_{x} & 0 & 0 & 0\\ 0 & f_{y} & 0 & 0\\ 0 & 0 & 1 & 0 \end{array}\right]\left[\begin{array}{c} x_{g,rh}\\ y_{g,rh}\\ z_{r,rh}\\ 1 \end{array}\right],i_{d,rh}=\frac{wi_{d,rh}}{w},j_{d,rh}=\frac{wj_{d,rh}}{w}$$

where ${\tilde{d}}_{x}=\left(\frac{w}{2}+d_{x}\right)\frac{1}{n_{x}}$, ${\tilde{d}}_{y}=\left(\frac{h}{2}-d_{y}\right)\frac{1}{n_{y}}$, $f_{x}$, $f_{y}$ are the focal lengths along the *x* and *y* axes, $n_{x}$, $n_{y}$ the pixel size, *w*, *h* the image sensor size, and $d_{x}$, $d_{y}$ the coordinates of the point of intersection of the camera optical axis with its sensing plane.

The method MapCameraPointToDepthSpacefrom class CoordinateMapper available in the Kinect for Windows v2 Windows Runtime API has been used [57]. It uses the intrinsic parameters of the camera determined during the factory calibration. If another sensor is used, whose software does not offer such functionality, the camera calibration process should be performed [58]. Left hand position $P_{d,lh}={\left[i_{d,lh},j_{d,lh}\right]}^{T}$ was determined in an analogous manner.

To segment hand in the depth image *D*, the following modified version of the seeded region growing algorithm was proposed (Algorithm 1):
**Algorithm 1:** Hand segmentation in the depth image using the modified version of the seeded region growing.
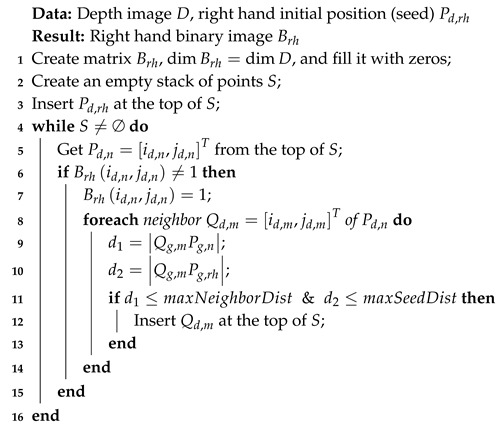


In Step 8, the 8-connectivity was used. The proposed modification consisted of transferring the decision from the image plane to the 3D space (Steps 9 and 10) and adding a new point to the hand when it was close enough to the adjacent point and not too far from the seed point (Condition 11). Transferring the decision to real-world coordinates reduced the method’s dependence on the distance between the hand and sensor. Equivalents of points $P_{d,n}$ and $Q_{d,m}$ in the reference system *g* were determined using the formulas:

(3) $$\begin{array}{c} x_{g}=\frac{\left(f_{x}+z_{g}\right)}{f_{x}}\left(\frac{W}{2}-i_{d}\right)n_{x}\\ y_{g}=\frac{\left(f_{y}+z_{g}\right)}{f_{y}}\left(\frac{H}{2}-j_{d}\right)n_{y}\\ z_{g}=\mu D\left(i_{d},j_{d}\right) \end{array}$$

where *W*, *H* are respectively the width and height of the image, $n_{x}$, $n_{y}$ denote the pixel size (mm/px), and μ is a scaling factor, which for the Kinect One sensor was equal to 1.

It was necessary to observe almost the entire silhouette of a person to recognize dynamic sign language expressions, so the user’s hand occupied a relatively small area in the image. Therefore, its shape was roughly described by the coefficient of compactness:

(4) $$\gamma_{rh}=\frac{P_{rh}^{2}}{4\pi S_{rh}}$$

and the eccentricity:

(5) $$\epsilon_{rh}=\frac{{\left(m_{rh,20}-m_{rh,02}\right)}^{2}+4m_{rh,11}^{2}}{S_{rh}^{4}}$$

were $P_{rh}$ is the surface area, $S_{rh}$ the circumference, and $m_{rh,pq}$ the central moment of the order pq [59] determined for the binary object corresponding to the right hand in the image $B_{rh}$. The slope of the main axis was also calculated:

(6) $$\phi_{rh}=0.5atan\frac{2m_{rh,11}}{m_{rh,20}-m_{rh,02}}$$

The binary image with the left hand $B_{lh}$ and the values $\gamma_{lh}$, $\epsilon_{lh}$, and $\phi_{lh}$ were determined analogously. When hands contacted or mutual occlusion was detected, $B_{rh}\cap B_{lh}\neq \emptyset$, the method used the values of compactness, eccentricity, and slope from the previous frame. In the case of contacts with another part of the body, the maxSeedDist parameter was used. Its value was determined during the calibration and was equal to the maximum distance between the hand’s center of gravity and its contour. Thanks to this, other points of the body, located further from the hand, were not included, even though they met the first condition given in Line 11 of Algorithm 1. This approach did not ensure an accurate separation of the hand, but with a rough description of the shape, it seemed to be sufficient.

The following feature vector was proposed:

(7) $$v=\left[x_{l,rh},y_{l,rh},z_{l,rh},\gamma_{l,rh},\epsilon_{l,rh},\phi_{l,rh},x_{l,lh},y_{l,lh},z_{l,lh},\gamma_{l,lh},\epsilon_{l,lh},\phi_{l,lh}\right]$$

**Figure 2:**
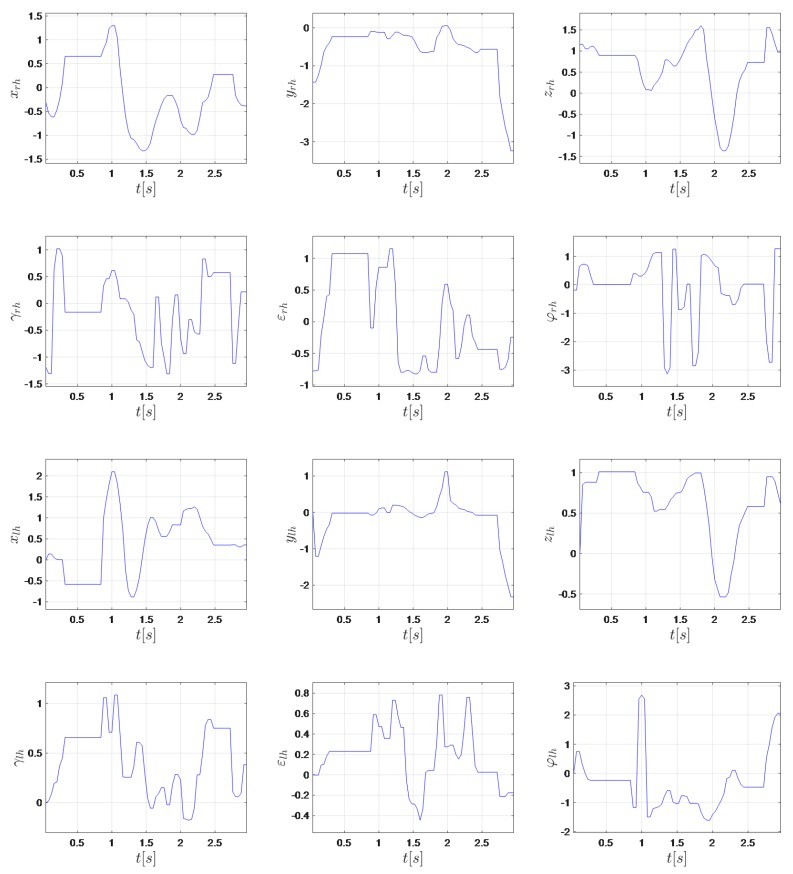
Time series corresponding to the expression “When the ID card is ready?” presented in Polish Sign Language (PSL).

Linguistic studies on sign languages revealed that each sign has three distinctive phonological features: handshape, location, and movement [60]. In the proposed approach, γ, ϵ, and ϕ describe handshape and *x*, *y*, and *z* the location, time series, and movement. Data were standardized to have a mean of 0 and a standard deviation of 1. Time series corresponding to the expression “When the ID card is ready?” presented in PSL are shown in Figure 2.

## 5. The Classifiers

The classification was carried out using: (1) the k-nearest neighbors (k-NN) algorithm [61], (2) hidden Markov models (HMM) [62], and (3) bidirectional long short-term memory (BiLSTM) [63]. For k-NN, time series were compared using the dynamic time warping (DTW) method [64].

### 5.1. DTW

To compare time series corresponding to expressions of different lengths, we used DTW [65]. It nonlinearly maps one sequence to another by minimizing the distance between them and compares similar series that are locally out of phase by time scale extension of compression. Users performed some parts of gestures representing the same expression with different velocities, so this advantage was especially important. For two time series $Q=\{q\left(1\right),q\left(2\right),\cdots ,q\left(T_{q}\right)\}$ and $R=\{r\left(1\right),r\left(2\right),\cdots ,r\left(T_{r}\right)\}$, a $T_{q}\times T_{r}$ matrix was considered, where the (i,j) element of the matrix contained the distance d(q(i),r(j)) between two points q(i) and r(j). A warping path, $W=w_{1},w_{2},\cdots ,w_{K}$, where $max\left(T_{q},T_{r}\right)\leq K\leq T_{q}+T_{r}-1$, is a set of matrix elements’ indexes $w_{k}=\left(i_{k},j_{k}\right)$ that satisfies three constraints: boundary condition, continuity, and monotonicity. The boundary condition constraint requires $w_{1}=\left(1,1\right)$ and $w_{K}=\left(T_{q},T_{r}\right)$. The continuity constraint limits the allowed steps to adjacent cells. The monotonicity forces the monotonic arrangement of points on the warping path. The warping path that has the minimum distance $d_{DTW}=\sum_{k=1}^{K}\frac{d(w_{k})}{K}$ between the two series is of interest, where $d\left(w_{k}\right)=d\left(q\left(i_{k}\right),r\left(j_{k}\right)\right)$. It is estimated using dynamic programming. To prevent mapping a relatively small section of one sequence to a much larger section of another one and to speed up the computation, the warping window constraint was applied [66]. It consists of defining a narrow strip around the diagonal connecting points $w_{1}$, $w_{K}$.

**Figure 3:**
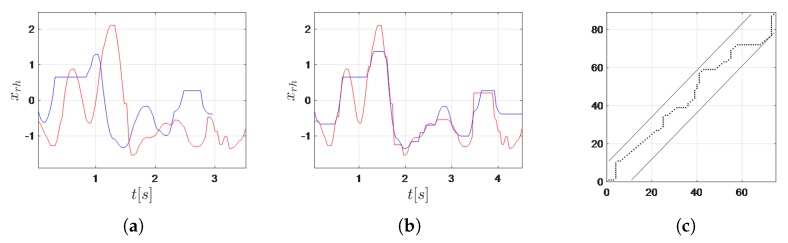
DTW of two time series corresponding to the expression “When the ID card is ready?” presented in PSL for the feature $x_{rh}$: (**a**) original time series, (**b**) time series after alignment, and (**c**) warping path with the warping window constraint.

As a result of the experiments, the city block metric was chosen, and the warping window constraint was set to 20 (Figure 3) [67].

The tests were carried out for the number of neighbors k=1,3,5.

### 5.2. HMM

Gestures’ executions are not perfect and may vary in speed and accuracy depending on mood or purpose. The human performance involves two distinct stochastic processes: immeasurable mental states and resultant actions that are measurable. Therefore to recognize dynamic gestures, we investigated hidden Markov models because they also consist of two stochastic processes. One of them was an unobservable Markov chain with a finite number of states, an initial state probability distribution, and a state transition probability matrix. The other one was a set of probability density functions associated with observations generated by each state. The model training consisted of an estimation of its parameters with the help of observation sequences. The expectation-maximization method (the Baum–Welch technique) can be used [62]. In the recognition step, the Viterbi algorithm was used to identify the class represented by a model that gave the highest probability of generating a tested sequence.

**Figure 4:**
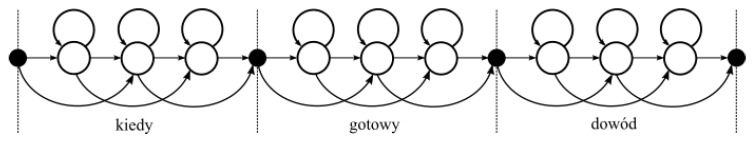
Bakis model for the expression “When the ID card is ready?” presented in PSL.

The expression model was composed of a series of connected models corresponding to individual words. Bakis models were used. The number of emitting states per word and emission probability distributions were determined experimentally, separately for training on original and extended data (see Section 6.2). In the first case, the number of states per word was equal to 3, and the unimodal distribution of observations was chosen based on the results for the number of states from 1–5 and uni- and bi-modal observations (Figure 4).

In the case of extended data, the number of states per word was equal to 9, and the unimodal distribution of observations was selected on the basis of experiments with the number of states being 3–13 and uni- and bi-modal observations.

### 5.3. BiLSTM

**Figure 5:**
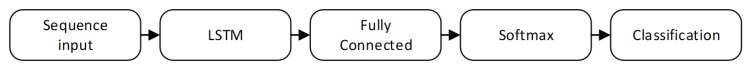
LSTM network architecture.

The LSTM classifier in the BiLSTM [68] version was used. The BiLSTM network is a modification of the long short-term memory (LSTM) network. The LSTM, first used by Hochreiter and Schmidhuber in 1997, is capable of learning long-term dependencies and is especially appropriate for the classification of time series. It has the chain structure shown in Figure 5 [69].

**Figure 6:**
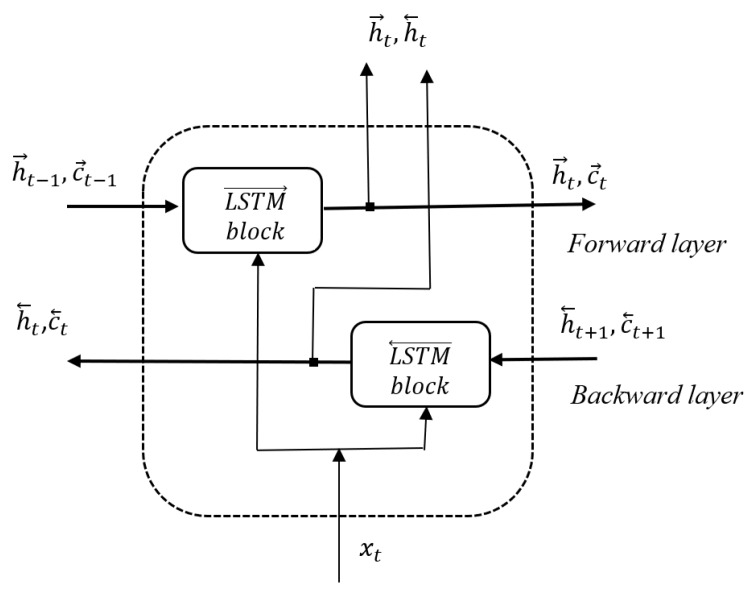
BiLSTM flow of data at time step *t*.

The sequence input layer introduces the data sequence or time series, and the LSTM layer learns the long-term relationships between the sequence time steps with its sophisticated structure, which consists of a set of recurrently connected memory blocks, each with one memory cell and three multiplicative gates: input, output, and forget gates. The gates control the long-term learning of sequence patterns. Each one is regulated by the sigmoid function, which learns during the training process, when to open and close, i.e., when to remember or forget information [69,70]. The network ends with a fully connected layer, a softmax layer, and a classification output layer to predict class labels. Unidirectional LSTM only preserves the information of the past because the only inputs it has seen are from the past. BiLSTM runs the inputs in two ways, one from past to future and one from future to past. This means that for every point in a given sequence, the BiLSTM has complete, sequential information about all points before and after it. The flow of data at time step *t* is shown in Figure 6.

The hidden state $\left({\vec{h}}_{t},{\overset{\leftarrow }{h}}_{t}\right)$ is the output of the BiLSTM layer at time step *t*. The memory cell state ${\vec{c}}_{t-1}$ (${\overset{\leftarrow }{c}}_{t+1}$) contains information learned from the previous (subsequent) time steps. At each time step *t*, the forward layer and the backward layer add information to or remove information from the respective cell state, based on the actual step of the sequence $x_{t}$. The layers control these updates using gates, as mentioned earlier. The BiLSTM network architecture is like that in Figure 5 with LSTM replaced by BiLSTM. It is especially useful when there is a need to teach the entire time series at every step. The BiLSTM usually learns faster than a one-directional approach, although it depends on the task. In our research, using BiLSTM was justified, because sign language expressions are sequences of interrelated elements. The hyperparameters were selected experimentally (see Section 6.4).

## 6. Experimental Results

### 6.1. Dataset and Tools

One-hundred-twenty-one Polish Sign Language expressions used to submit an application for an ID card or collect an ID card were recognized. Time series were obtained from recordings prepared in the SyKoMi system, as described in Section 4. Each expression was shown five times by four people, A, B, C, and D. Among them were two women and two men. Their age ranged from 20 to 63 years, height from 162–189 cm, and weight from 62 to 108 kg. The minimum number of words was 1, maximum 8, and average 3. The data acquisition frequency was 25 frames per second. The shortest expression lasted 0.960 and the longest 10.240 seconds. The average length was 3.327 seconds.

Expressions were divided into seven groups corresponding to thematic threads, which could be distinguished in typical conversation schemes. Group numbers, their descriptions, and sizes are given in Table 1.

The following tools were used: MS Visual C++ 2015 (data acquisition and processing), MATLAB R2019b (DTW and BiLSTM), and the Hidden Markov Model Toolkit 3.4.1 (HMM) [71]. The experiments were carried out on a computer with an i7-6820HQ 2.7 GHz processor and 32 GB RAM.

### 6.2. Data Augmentation

The training set (set of patterns) was augmented with artificially generated data. Each pair of original time series related to the same expression was aligned using DTW and used to create three new ones according to the formula:

(8) $$c=\tau a\left(a_{s}\right)+\left(1-\tau \right)b\left(b_{t}\right)$$

where *a*, *b* are the original time series, *c* the result series, $a_{s}$, $b_{t}$ the indexes determining the “warping path” for *a* and *b*, and τ=−0.1,0.5,1.1.

During the experiments, the leave-one-subject-out (l-o-s-o) protocol was adopted, i.e., sign expressions performed by a specific person were recognized by the classifier trained on data from remaining people. Thus, one expression had 5×3=15 original utterances in the training set. From 15 original time series, two could be selected in 105 ways, so 105×3=315 artificial time series for one expression were obtained. One expression in the training set was represented by original data and artificial data, i.e., a total of 15+315=330 utterances.

### 6.3. Results

**Figure 7:**
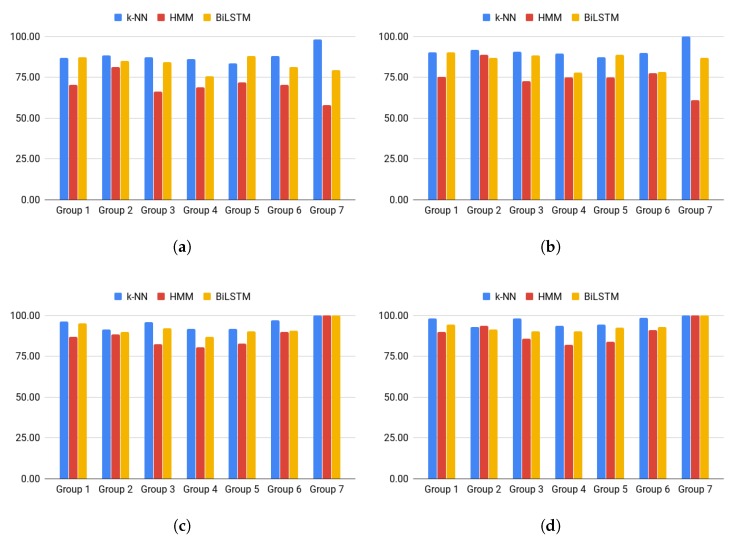
Recognition rates: (**a**) ACOD, (**b**) GOD, (**c**) ACED, (**d**) GED.

Three classifiers were used: (1) k-NN, (2) HMM, and (3) BiLSTM. Four variants were tested: ACOD, ACED, GOD, GED. OD and ED mean original and extended data, respectively. AC indicates that classifiers were trained using all classes. G means that classifiers were trained separately for each group. For G, the recognition rate of expressions from a given group was taken into account while assessing the classifier, and for the AC recognition rate, all classes were tested. To compare both variants, the results for all classes were decomposed into appropriate groups. The obtained results are presented in Table 2 and Figure 7.

Classifier response times are given in Table 3.

For the ACOD problem, the best results were obtained for k-NN (five of seven groups). BiLSTM was the best for the other two groups. In all seven cases, the worst results were obtained for HMM. It turned out that models with three states per word were insufficient to precisely model the recognized expressions.

After considering extended data (ACED problem), k-NN turned out to be the best classifier in six out of seven cases. Only for Group 5, the best result was obtained for BiLSTM. However, it is worth noting the significant increase in the response time of k-NN, which must compare a recognized sequence with each pattern from the extended dataset. Considering this, BiLSTM may be the best option. In most cases, an increase in recognition rates was observed compared to the variant based only on original data (ACOD). Depending on the group, kNN results improved from around 1.97 to 4.00 percentage points. For HMM, the improvement was much more significant, ranging from 3.00 to 7.58 points. It was possible to train larger models thanks to the extended data available. In the case of BiLSTM, results were better by 1.00 to 7.51 points, except for Group 6, for which the result was 3.22 points worse.

After division into groups (GOD problem), a significant increase in recognition efficiency was observed compared to ACOD. Depending on the group, it ranged from 1.87 to 9.31 points (k-NN), 7.13–41.87 points (HMM), and 2.50–20.63 points (BiLSTM). In six out of seven cases, the k-NN classifier was the best. For Group 7, 100% recognition rates were obtained for all classifiers.

After considering extended data, when training took place only on utterances belonging to the recognized group (GED), an increase in recognition rates was observed by 1.43 to 2.50 points (k-NN) and 1.00 to 5.15 points (HMM). For BiLSTM, recognition rates were better by 1.37 to 3.26 points for four of the seven groups. For Groups 1 and 3, there was a decrease in recognition rates by 0.52 and 1.58 points, respectively. In the GED problem, in five out of six cases, the k-NN classifier turned out to be the best. For Group 2, the best result was obtained for HMM, while for Group 7, all classifiers reached 100%.

### 6.4. BiLSTM Hyperparameters’ Selection

BiLSTM network parameters were selected experimentally (Table 4, Table 5 and Table 6).

The dropoutProbability and initialLearnRate were selected using the grid search approach through a specified discrete subset of parameters. The l-o-s-o results were used as a performance metric. Values obtained for the ACOD problem are shown in Table 5. In the same way, the selection of parameters for other problems was carried out (Table 6).

### 6.5. Comparison with Other Works

The comparison of our work with recent papers on dynamic sign language recognition using RGB-D data is given in Table 7.

The proposed solution was practically oriented; therefore, it had some advantages over other works.

1. Only four papers mentioned the involvement of the deaf in a dataset’s preparation. In our case, deaf people participated not only in the data collection, but also during the design of the user interface and verification of the system in a laboratory and real conditions. It is necessary to create a useful application for people with disabilities.
2. The vocabulary considered in this work concerned a specific application from a very narrow field. With the current state of knowledge about sign language recognition, only this approach guaranteed reliability that was acceptable in a real system. Other works concerned broader domains (e.g., daily activity) requiring vocabularies larger than the considered one to ensure consistent communication, or they focused on unrelated words.
3. Only two of the compared works concerned the recognition of signed expressions. However, the number of considered classes was smaller than in our case.
4. In all compared works except one, recognized words or expressions were extracted manually from the video stream. In our solution, a simple but effective algorithm for automatic expression spotting was employed.
5. More than half of the work used only skeletons. Therefore, these methods cannot be applied for gestures, for which the shape of the hand is the only distinctive feature.
6. Most works, especially those with better results, did not use the leave-one-subject-out (l-o-s-o) protocol, which allowed for the more accurate evaluation of the method.

## 7. Conclusions

The paper discussed the recognition of dynamic PSL expressions in an experimental system supporting deaf people in an office when applying for an ID card. A method of processing a continuous stream of RGB-D data recorded by the Kinect sensor and feature vector inspired by linguistic research was proposed. The classification was tested using three methods most commonly used to recognize dynamic gestures: k-NN, HMM, and BiLSTM. The l-o-s-o protocol was used for the dataset containing 121 PSL expressions performed five times by four people. Since the data collection process was a burdensome task and required the involvement of deaf people, the data augmentation method was also tested. Because the identified conversation patterns between an office clerk and a deaf person could be divided into compact thematic threads, the considered expressions were divided into inseparable groups, and the effectiveness of recognition in individual groups was examined. This allowed for higher recognition rates and shorter response times, which was important in the implemented system, especially for the k-NN classifier. In the current version of the system, switching between these groups was done by the office clerk, who was a conversation moderator.

The installation of the system in the office was experimental. However, it allowed gathering valuable experience. Preliminary tests of the system in real conditions showed that further work was needed, not only in the area of gesture recognition. Thanks to the use of the RGB-D sensor, the number of restrictions imposed on the deaf person significantly decreased, but the hands still should be kept on the knees in the intervals between utterances. It turned out that this was not an easy task for people for whom hands are the primary means of expression. In the future, therefore, it will be necessary to develop a better gesture spotting system, as well as a method of distinguishing intentional utterances from involuntary gestures and expressions from classes not belonging to the dictionary under consideration. With the current state of knowledge, solving this problem in a way that can be used in a practical system is still a big challenge [1].

Deaf people, especially the elderly, are afraid of modern technology. Therefore, it is necessary to increase their involvement not only in the process of collecting training datasets, but also in other stages of the software development and maintenance cycle. Their participation in the phase of user interface design and system evaluation is particularly important. Training on the use of the system by deaf people is also necessary.

The system was dedicated, and its transfer or extension to another domain is a time-consuming and costly process. Recording extensive datasets and training classifiers are necessary. There are different sign language dialects in the country, which is an additional difficulty. Many words have their local equivalents or several interchangeable forms. Therefore, deaf people from a given region must participate in the dataset recording process.

It is also necessary to enrich the training set constantly, taking into account its diversity in terms of gender, age, clothing, lighting, and background. Moreover, further research in sign language linguistics should contribute to the development of better classifiers that increase system flexibility. However, the undeniable positive effect that has already been observed is a better understanding of deaf people’s needs by office clerks operating the system.

## Figures and Tables

**Table 1:** Division of expressions into groups.

Group Number	Description	Number of Expressions
I	Welcome and explanation of the reason for the visit	29
II	The reason for applying for an ID card	33
III	Application form	19
IV	Identity document	23
V	Photo	10
VI	Receiving ID card	14
VII	Fee	8

**Table 2:** Recognition rates (%). AC, all classes; G, group; OD, original data; ED, extended data.

Classifier	Problem	Group 1	Group 2	Group 3	Group 4	Group 5	Group 6	Group 7
k-NN	ACOD	86.90	88.48	87.37	86.30	83.50	88.21	98.13
	ACED	90.52	91.82	90.79	89.57	87.50	90.00	100.00
	GOD	96.21	91.36	96.05	91.96	92.00	97.14	100.00
	GED	98.10	93.03	98.42	93.70	94.50	98.57	100.00
HMM	ACOD	70.52	81.36	66.32	68.91	72.00	70.36	58.13
	ACED	75.52	88.94	72.63	75.00	75.00	77.50	61.25
	GOD	87.07	88.49	82.37	80.44	83.00	90.00	100.00
	GED	89.83	93.64	85.79	81.96	84.00	91.07	100.00
BiLSTM	ACOD	87.41	85.00	84.21	75.65	88.00	81.43	79.37
	ACED	90.34	86.82	88.68	78.04	89.00	78.21	86.88
	GOD	95.17	90.15	92.11	86.96	90.50	90.71	100.00
	GED	94.65	91.52	90.53	90.22	92.50	92.86	100.00

**Table 3:** Classifier response times (s).

Problem	k-NN	HMM	BiLSTM
ACOD	0.200	0.120	0.001
ACED	3.500	0.260	0.001
GOD	0.055	0.080	0.002
GED	0.860	0.110	0.002

**Table 4:** Hyperparameter values in BiLSTM network training.

Parameter	Value
Size of the mini-batch (miniBatchSize)	15 (OD), 330 (ED)
Number of hidden units (numHiddenUnits)	150
Maximum number of epochs (maxEpochs)	100
Regularization factor (l2Regularization)	0.0001
Probability to drop out input elements (dropoutProbability)	Table 5
Initial learning rate (initialLearnRates)	Table 5
Learning rate schedule (learnRateSchedule)	“none”
Number of epochs for dropping the learning rate (learnRateDropPeriod)	10
Factor for dropping the learning rate (learnRateDropFactor)	0.1
Solver for training network (solverName)	“Adam”

**Table 5:** An example of dropoutProbability and initialLearnRate selection in the ACOD problem.

DropoutProbability	InitialLearnRates	Recognition Rate (%)
0.1	0.0005	82.40
0.1	0.001	82.52
0.1	0.005	81.49
0.2	0.0005	81.24
0.2	0.001	81.57
0.2	0.005	80.04
0.3	0.0005	80.00
0.3	0.001	83.39
0.3	0.005	82.40

**Table 6:** DropoutProbability and initialLearnRate hyperparameters.

	DropoutProbability		InitialLearnRates	
	**OD**	**ED**	**OD**	**ED**
AC	0.3	0.3	0.001	0.001
G1	0.3	0.1	0.001	0.005
G2	0.2	0.1	0.001	0.005
G3	0.3	0.2	0.005	0.001
G4	0.2	0.3	0.001	0.005
G5	0.3	0.2	0.005	0.001
G6	0.2	0.3	0.001	0.005
G7	0.2	0.3	0.001	0.001

**Table 7:** Comparison with other works. l-o-s-o, leave-one-subject-out.

Work	Year	Dataset	Deaf	Vocabulary	Modality	Gesture	Classifier	Recognition	Protocol
			Involvement	Domain		Spotting		Rate	l-o-s-o
[72]	2020	Chinese SL	n/a	daily life	skeleton	manual	Bi-LSTM	82.55%	no
		500 words							
		50 users							
		5 repetitions							
[73]	2019	Indian SL	n/a	words describing	RGB	manual	CNN	89.69%	yes
		200 words		human actions	depth				
		10 users							
		10 repetitions							
[74]	2018	Indian SL	n/a	different	skeleton	manual	HMM	83.77%	yes
		30 words		unrelated words					
		10 users							
		9 repetitions							
[75]	2017	Chinese SL	n/a	n/a	skeleton	manual	Adaptive	67.34%	yes
		370 words			RGB		HMM		
		5 users							
		5 repetitions							
[76]	2017	Mexican SL	yes	greetings (4)	skeleton	manual	DTW	98.57%	no
		20 words		questions (2)					
		35 users		family (5)					
		1 repetition		pronouns (2)					
				places (2)					
				others (2)					
[77]	2016	Chinese SL	yes	n/a	skeleton	manual	Adaptive	98.80%	no
		500 words			RGB		HMM		
		1 user							
		5 repetitions							
[78]	2016	Chinese SL	n/a	daily life	skeleton	manual	HMM	82.70%	no
		100 words							
		50 users							
		5 repetitions							
[79]	2016	Chinese SL	n/a	daily	skeleton	manual	HMM	88.00%	no
		21 words		communication					
		8 users							
		20 expressions							
		2 users							
[80]	2015	American SL	n/a	selected signs	skeleton	manual	Latent	82.90%	n/a
		73 words		used by	RGB		SVM		
		63 expressions		beginning signers					
		10 users							
		3 repetitions							
[81]	2015	Indian SL	yes	different	skeleton	automatic	SVM	86.16%	no
		37 words		unrelated words					
		15 users							
		5 repetitions							
[82]	2015	Arabic SL	yes	words that can be	skeleton	manual	HMM	64.61%	yes
		16 words		used in hospital	RGB				
		4 users							
		3 repetitions							
**our**	**2020**	**Polish SL**	**yes**	**submitting**	**skeleton**	**automatic**	**k-NN**	**98.57%**	**yes**
		**121 expressions**		**application**	**depth**		**HMM**	**91.07%**	
		**4 users**		**for ID card**			**BiLSTM**	**92.86%**	
		**5 repetitions**							
